# Endothelial cell-derived Apelin inhibits tumor growth by altering immune cell localization

**DOI:** 10.1038/s41598-021-93619-5

**Published:** 2021-07-07

**Authors:** Liuying Hu, Yumiko Hayashi, Hiroyasu Kidoya, Nobuyuki Takakura

**Affiliations:** 1 Department of Signal Transduction, Research Institute for Microbial Diseases, Osaka University, 3-1 Yamada-oka, Suita, Osaka 565-0871 Japan; 2 World Premier Institute Immunology Frontier Research Center, Osaka University, Suita, Japan; 3 Integrated Frontier Research for Medical Science Division, Institute for Open and Transdisciplinary Research Initiatives (OTRI), Osaka University, Suita, Japan

**Keywords:** Cancer, Cancer microenvironment, Tumour angiogenesis

## Abstract

The Apelin/APJ signalling pathway, involved in multiple physiological and pathological processes, has been attracting increasing interest recently. In our previous study, Apelin overexpression in colon26 tumor cells suppressed tumor growth by inducing vascular maturation. Here, we found that MC38 and LLC tumor growth were greater in the absence of Apelin than in wild-type (WT) mice, suggesting that Apelin acts as a tumor suppressor. Consistent with this, treating WT mice with [Pyr^1^]Apelin-13 inhibited tumor growth. In MC38 tumors, only endothelial cells (ECs) strongly express APJ, a cognate receptor for Apelin, indicating that EC-derived Apelin might regulate tumor formation in an autocrine manner. Comparing with WT mice, larger numbers of vessels with narrower diameters were observed in tumors of Apelin knockout mice and lack of Apelin enhanced tumor hypoxia. Investigating immune cells in the tumor revealed that [Pyr^1^]Apelin-13 infusion induced the accumulation of CD8^+^ and CD4^+^ T cells in central areas. Moreover, RNA-sequencing analysis showed that Apelin induces chemokine CCL8 expression in ECs. Thus, enhancing anti-tumor immunity might be one of the mechanisms by which Apelin is involved in tumor growth. Our result indicated that increased CCL8 expression might induce CD8^ +^  T cells infiltration into tumor and tumor inhibition.

## Introduction

Apelin is the endogenous ligand for APJ, a G-protein-coupled receptor with 7 transmembrane domains. The receptor is widely expressed on the surface of many types of cells, including cardiomyocytes, adipocytes, neuronal cells, endothelial cells (ECs), and others. The Apelin/APJ pathway appears to modulate many physiological processes and contribute to certain pathological conditions. It is especially important in blood vessel development by inducing EC proliferation and vascular maturation^1^. In pathological conditions, Apelin has been identified as a tumor endothelium-specific gene, not expressed by normal endothelium. In glioblastoma, both Apelin and APJ are highly upregulated in the microvasculature^2^, indicating that the Apelin/APJ system is involved in tumor angiogenesis. In a human non-small cell lung cancer xenograft model, overexpression of Apelin in cancer cells significantly stimulated tumor growth and increased micro-vessel densities and perimeters in vivo^3^. However, in our previous study, overexpression of Apelin greatly inhibited colon 26 tumor growth in mice after subcutaneous cancer inoculation by inducing tumor vascular maturation^4^. This finding suggested that Apelin might have diverse functions in tumor formation depending on the tumor microenvironment.

Regulation of tumor growth is closely associated with intra-tumoral immune cell functions. Marked accumulation of intra-tumoral CD8^+^ T cells correlates with better prognosis whereas accumulation of macrophages or neutrophils in the tumor is often associated with a worse prognosis^5,6^. Therefore, immune cell infiltration is considered as one of the pivotal indexes that control tumor growth. Thus, one variable affecting tumor growth is the presence and function of tumor-associated T cells, especially CD8^+^ cytotoxic T cells^7^. An obvious requirement for successful T cell-mediated tumor control is for the T cells to be able to enter the tumor^8^. However, low levels of infiltration into central areas of the tumor limits the ability of T cells to mediate their necessary anti-tumor cytotoxic functions. Our recent study demonstrated that a mature and functional tumor vasculature helps circulating T cells to migrate into central areas of the tumor more effectively^9^.

T cell infiltration into the tumor is controlled by a coordinated sequence of rolling, adhesion, and transmigration steps of T cells in order to extravasate into areas where they are needed^10^. Crucial factors for T cell infiltration are adhesive interactions between T cells and endothelial surfaces, and integration of chemokine-mediated signalling^11^. It is widely accepted that infiltration of circulating CD8^+^ T cells into tumors is governed by adhesion molecules and chemokines expressed by the ECs of the vasculature^12^. Although Apelin/APJ signalling was suggested to induce the formation of focal adhesions^13^, the expression on ECs of several adhesion molecules involved in T cell homing exhibited no significant differences between wild-type (WT) and Apelin-knockout (KO) mouse tumors.

In the present study, we suggest that Apelin’s ability to inhibit tumor growth depends on the particular tumor model studied. We used genetically-engineered mice (Apelin-KO) or animals continuously infused with [Pyr^1^]Apelin-13. The MC38 colon cancer cell subcutaneous inoculation model was found suitable for analysing the tumor suppressive function of Apelin. Using this model, we determined how Apelin affects tumor vascularity, focusing on structure and function, and in particular, immune cell infiltration. To analyse transcriptional changes induced by Apelin in ECs, RNA sequencing was performed to discern biological differences between tumor ECs isolated from WT and Apelin-KO mice. We found that CCL8 was more highly expressed in tumor ECs from WT than Apelin-KO mice. CCL8 is a chemoattract protein belonging to the CC chemokine family and is involved in the recruitment of resting and activated T cells as well as other immune cells into tissues^14–16^. Therefore, we analysed CCL8 receptor expression in the tumor microenvironment and consider how the Apelin/CCL8 axis is involved in tumor immunity.

## Results

### Apelin inhibits tumor growth in mouse models

**Figure 1:**
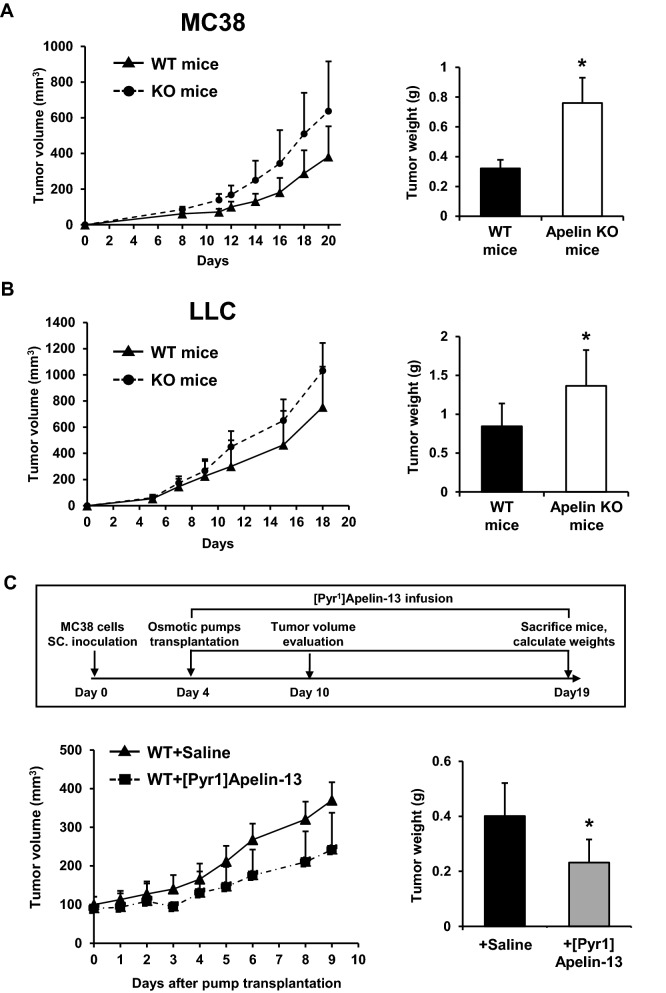
Effects of Apelin on tumor volume and weight. (**A**) Tumor volume curves and tumor weight of MC38 cancer cells subcutaneously (s.c.) inoculated into WT or Apelin-KO mice (n = 7 for each group). (**B**) Tumor growth curves and tumor weight of LLC cells s.c. inoculated into WT or Apelin-KO mice (n = 6 for each group). (**C**) Schedule of tumor models using ALZET osmotic pumps to infuse [Pyr^1^]Apelin-13 or saline, and MC38 tumor growth curves (n = 5 for each group). MC38 tumor weights on day 19 after inoculation were calculated (n = 5 for each group). All experiments were repeated at least twice. The error bars indicate mean ± SD and all data were analyzed by two-sided Student's t-test. *p < 0.05.

In our previous study, overexpression of Apelin greatly inhibited colon 26 tumor growth in subcutaneous inoculation models^4^, suggesting that it might play an important role in tumor progression. Here, to determine the mechanism whereby endogenous Apelin regulates tumor growth, C57BL/6 WT and Apelin-KO mice (C57BL/6 background) were subcutaneously inoculated with murine colon carcinoma MC38 cells or Lewis lung carcinoma LLC cells. In both tumor models, although tumor growth seemed to be accelerated, there are no significant difference of tumor volume. However, tumor growth in both tumor models was enhanced significantly in Apelin-KO mice (Fig. 1A,B). We think that tumor weight is more accurate than tumor volume. Furthermore, depletion of Apelin thus enhances tumor growth, most noticeably MC38. Therefore, we selected the MC38 tumor model to analyse the function of Apelin in further studies.

To assess whether higher serum concentration of Apelin can inhibit tumor growth, ALZET osmotic pumps were employed to infuse [Pyr^1^]Apelin-13 polypeptide continuously into tumor-bearing mice for 2 weeks. Compared with the saline control (WT + Saline), the MC38 tumor exhibited decreased growth in mice infused with Apelin (WT + [Pyr^1^]Apelin-13) (Fig. 1C). [Pyr^1^]Apelin-13 infusion may reverse the phenotype observed in Apelin-KO mice. Therefore, we infused [Pyr^1^]Apelin-13 into Apelin-KO mice that had been inoculated with MC38 tumor cells. However, tumor growth in animals infused with [Pyr^1^]Apelin-13 or saline was not significantly different (Fig. S1). It is possible that Apelin deficiency during embryogenesis and after birth may induce aberrant expression of thus-far unidentified factors affecting Apelin sensitivity. Further precise investigations in terms of Apelin sensitivity of Apelin-deficient mice are required to resolve this issue in future.

Thus, Apelin showed tumor growth inhibitory activity in mice, especially in these MC38 and LLC subcutaneously inoculated tumor models. Other publications have reported that Apelin has tumor-promoting effects, but this disparity might be due to different tumor microenvironments depending on the cancer cell type used in the models.

### Endothelial cell-induced Apelin regulates tumor growth via the Apelin/APJ axis

**Figure 2:**
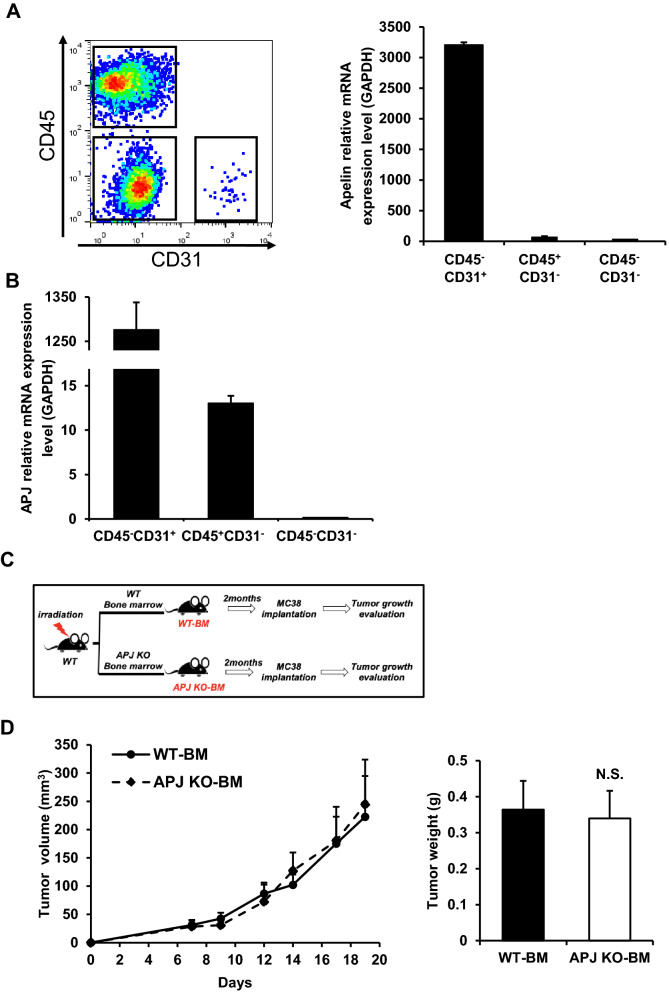
Evaluation of Apelin and APJ expression and involvement of APJ^+^ immune cells in MC38 tumor formation. (**A**) Cells in the MC38 tumor microenvironment at day 19 of tumor cell inoculation were separated into 3 groups by flow cytometry, i.e., CD45+ CD31−, CD45−CD31+ and CD45−CD31− cells. Apelin expression levels were evaluated by qRT-PCR and normalized against GAPDH. (**B**) APJ expression levels were evaluated by qRT-PCR and normalized against GAPDH. (**C**) Schedule of tumor models using WT or APJ-KO bone marrow-transplanted mice (n = 10 for each group). MC38 tumor growth curves were measured until day 19. (**D**) MC38 tumor weights on day 19 after MC38 cell inoculation were calculated (n = 10 for each group). All experiments were repeated at least three times. The error bars indicate mean ± SD and all data were analyzed by two-sided Student's t-test.

Generally, APJ is expressed in different lineages originating from the mesoderm, such as the hemopoietic and endothelial lineages^1^. To further investigate which type of cells secrete Apelin in MC38 tumor-bearing mice, cells from the tumor microenvironment were examined by flow cytometry. Of the CD45^+^CD31^−^ hematopoietic cells, CD45^−^CD31^+^ ECs and CD45^−^CD31^−^ non-hematopoietic and non-endothelial cells, only the ECs expressed very high levels of Apelin^4^. This was confirmed by qRT-PCR (Fig. 2A) and indicates that ECs are the major source of Apelin affecting tumor growth in this model.

Apelin is an endogenous ligand of the G-protein-coupled APJ receptor. It has been reported that Apelin is involved in cell proliferation, migration, and adhesion by binding to APJ, a G-protein-coupled receptor with 7 transmembrane domains^17^. Therefore, we measured the level of expression of APJ in the 3 cell populations as above, using qRT-PCR. CD45^−^CD31^+^ cells, representing vascular ECs, expressed APJ at a high level, while CD45^+^CD31^−^ cells also were only weakly positive for APJ (Fig. 2B).

Although the level of APJ expression was lower than in ECs, CD45^+^CD31^−^ hematopoietic-derived tumor-associated immune cells are suggested to affect tumor progression to some extent via their cytotoxic effects (CD8^+^ T cells) or their immunosuppressive action (regulatory T cells). In order to clarify whether APJ-expressing immune cells influence tumor growth, mice depleted of APJ in bone marrow cells were generated via BM transplantation (Fig. 2C). WT recipient mice were first lethally irradiated to ablate their hematopoietic system. BM cells from either WT or APJ-KO mice were then transferred to recipients via tail vein injection. Later, MC38 cells were inoculated subcutaneously into recipient mice after their hematopoietic system had been reconstituted by donor BM cells. According to evaluations of tumor size and weight, tumor growth was not significantly different in mice transplanted with WT (WT-BM) or APJ-KO (APJ KO-BM) BM cells (Fig. 2C,D). This excludes the possibility that Apelin exerts a direct effect on immune cells responding to the tumor. Collectively, therefore, these data imply that Apelin regulates tumor growth through an Apelin/APJ autocrine pathway in ECs or in a paracrine manner between neighbouring ECs.

### Apelin enlarges tumor vessel lumens and improves vascular function

**Figure 3:**
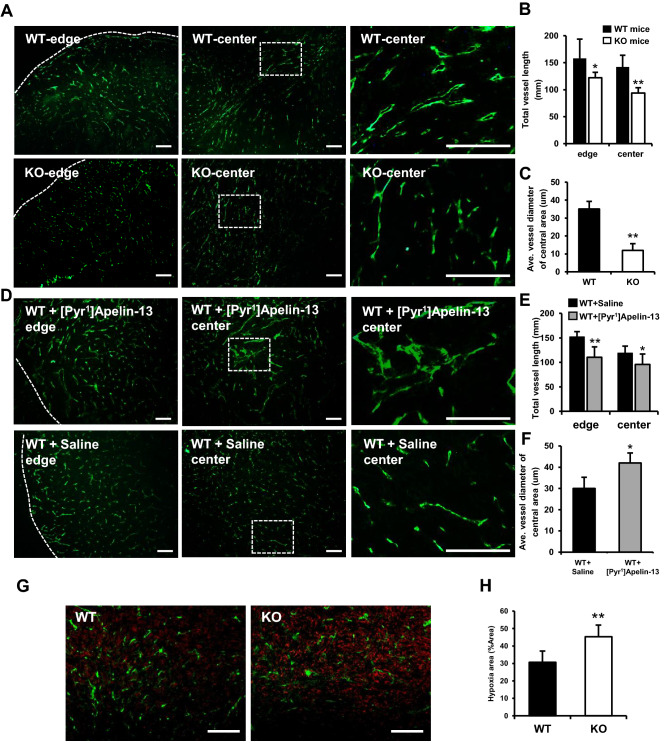
Tumor vessel formation and functional analysis. (**A**) Immunofluorescence staining of CD31 (green) in frozen sections of MC38 tumors from WT or Apelin-KO mice. Scale bar = 200 µm. (**B**) Quantification of total vessel length (6 random fields of 5 independent tumor sections). [edge; 158.05 ± 35.47 (WT) vs 122.14 ± 23.17 (KO), p = 0.04, center; 141.99 ± 21.99 (WT) vs 93.80 ± 13.07 (KO), p = 0.0003]. (**C**) Quantification of average vessel diameter of tumor central area (6 random fields of 5 independent tumor sections). (**D**) Immunofluorescence staining of CD31 (green) in frozen sections of MC38 tumors from WT mice receiving [Pyr^1^]Apelin-13 or saline infusions using osmotic pumps. Scale bar = 200 µm. (**E**) Quantification of total vessel length (6 random fields of 5 independent tumor sections). [edge; 152.10 ± 10.07 (Saline) vs 110.55 ± 21.27 ([Pyr^1^]Apelin-13), p = 0.0005, center; 118.93 ± 14.30 (Saline) vs 95.89 ± 21.04 ([Pyr^1^]Apelin-13), p = 0.03]. (**F**) Quantification of average vessel diameter of tumor central area (6 random fields of 5 independent tumor sections). (**G**) Immunofluorescence staining of CD31 (green) in MC38 tumor sections from WT and Apelin-KO mice. Hypoxic status was revealed by Hypoxyprobe-1 (red). Scale bar = 200 µm. (**H**) Quantification of the hypoxic area in MC38 tumor sections. Data are mean ± SD and were analyzed by two-sided Student's t-test. *p < 0.05, **p < 0.01.

The Apelin/APJ pathway modulates many physiological and pathological conditions or processes, especially vascular formation, via the induction of EC proliferation and vascular maturation. For instance, apelin was reported to induce enlargement of blood vessels in the dermis of apelin Tg mice, and overexpression of Apelin in colon 26 tumor cells significantly suppressed tumor growth by inducing tumor vascular maturation. To clarify how EC-secreted Apelin inhibits tumor growth in the MC38 model, first, we evaluated tumor vessel formation. Corresponding to Apelin function in normal tissues, WT mice exhibited longer tumor blood vessels relative to their Apelin-KO tumor-bearing counterparts (Fig. 3A,B). In addition, Apelin deficiency attenuated the vascular luminal space and induced merely cord-like structures of CD31-positive ECs in the central tumor area, different from tumors in WT mice (Fig. 3C). In tumors from [Pyr^1^]Apelin-13-infused mice (designated WT + [Pyr^1^]Apelin-13), total vessel length was shorter than in tumors from un-infused mice both at the edge and center of the tumor (Fig. 3D,E). The “edge” of the tumor was defined as within 1.5 mm of its margin, and the “center” was within 1.5 mm of its center. However, total vessel density was not significantly different between WT + [Pyr^1^]Apelin-13 and the WT + Saline control group (Fig. S2), suggesting that higher concentrations of [Pyr^1^]Apelin-13 might enhance vessel calibre enlargement rather than elongation. Quantification of vessel diameters in the central regions of WT + [Pyr^1^]Apelin-13 tumors are consistent with this hypothesis (Fig. 3F). Apelin deficiency but also administration of Apelin using [Pyr^1^]Apelin-13 both reduced vessel length in tumors. This may seem contradictory, but the Apelin-APJ system induces proliferation of endothelial cells and it is therefore reasonable that vascular length in the tumors of Apelin KO mice are reduced relative to WT mice. On the other hand, Apelin is also involved in the maturation of blood vessels and excessive amounts of Apelin may suppress vascular elongation of immature blood vessels in the tumor microenvironment. Thus, it is possible that both too little and too much Apelin reduce vascular elongation, but further precise analysis is required to clarify this.

Tumor vasculature is generally structurally immature and leaky, resulting in tumor tissue usually being hypoxic, especially in the central area. As far as is known, Apelin becomes a trigger for vessel maturation by facilitating cell–cell adhesion of ECs and modifying the caliber of the capillaries^18^. Therefore, to investigate whether Apelin also contributes to functional blood vessel formation in MC38 tumor models, the hypoxic status of central tumor regions was evaluated by hypoxyprobe-1. Apelin KO-mouse tumors also manifested greater hypoxia in the central region than in WT mice (Fig. 3G,H). Taken together, these results indicate that apelin-mediated elongation and enlargement of tumor vessels also induces their functional maturation in the MC38 model.

### The accumulation of CD8^+^ and CD4^+^ T lymphocytes in the central area of the tumor is enhanced by Apelin

Responses of CD8^+^ and CD4^+^ T cells against tumors are considered one of the principle mechanisms controlling tumor growth^19^. Accordingly, the infiltration of these cells into central areas of the tumor has been shown to have significant positive prognostic importance^8^. Along with the maturation of tumor vasculature, infiltration of T cells might also be altered by Apelin.

**Figure 4:**
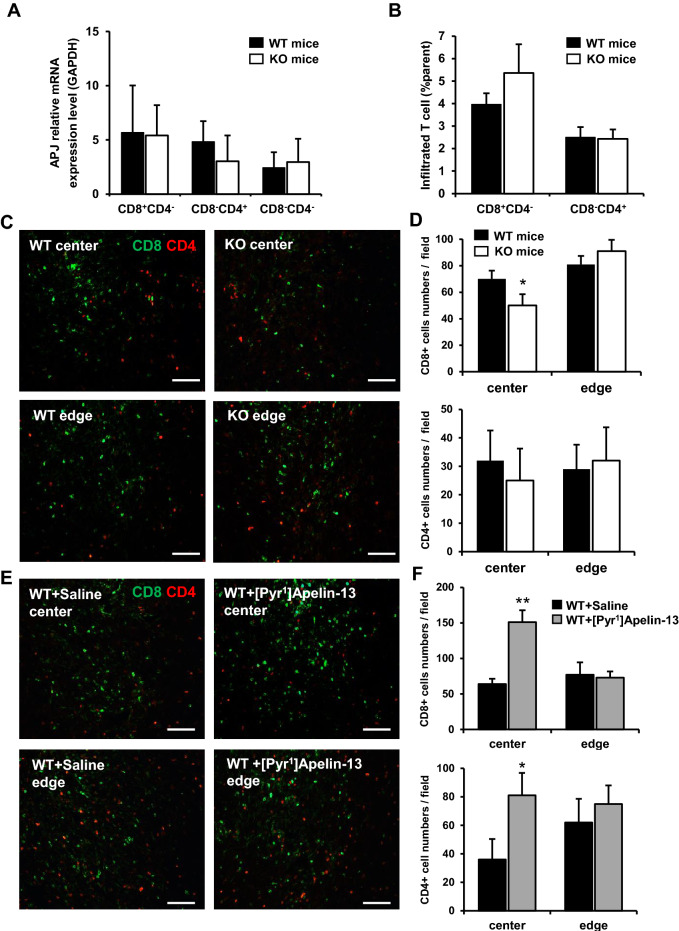
CD8^+^ and CD4^+^ T cell infiltration and accumulation in tumors. (**A**) APJ expression by qRT-PCR. CD8^+^ and CD4^+^ T cells were isolated from WT and Apelin-KO mice bearing tumors at day19 by flow cytometry. APJ expression levels were normalized to GAPDH. (**B**) Flow cytometric analysis of total infiltrated CD8^+^ and CD4^+^ T cells. Cell numbers are presented as percentages of total living cells (n = 6 for each group). (**C**) Immunofluorescence staining of CD8 (green) and CD4 (red) in the frozen sections of MC38 tumors from WT or Apelin-KO mice. Tumor samples were collected at day 19. Scale bar = 100 µm. (**D**) Quantitative evaluation of the number of infiltrated CD8^+^ and CD4^+^ T cells in MC38 tumors (6 random fields of 5 independent tumor sections). (**E**) Immunofluorescence staining of CD8 (green) and CD4 (red) in the frozen sections of MC38 tumors from WT mice infused with [Pyr^1^]Apelin-13 or saline using osmotic pumps. Tumor samples were collected at day 19. Scale bar = 100 µm. (**F**) Quantitative evaluation of the number of infiltrated CD8^+^ and CD4^+^ T cells in MC38 tumors (6 random fields of 5 independent tumor sections). The error bars indicate mean ± SD and all data were analyzed by two-sided Student's t-test. *p < 0.05, **p < 0.01.

As shown in Fig. 2B, APJ expression was observed in hematopoietic cells in the tumor microenvironment. Therefore, we isolated CD8^+^ and CD4^+^ T cells from MC38 tumors in WT mice and APJ-KO mice and compared APJ expression levels by qRT-PCR. However, no APJ expression was observed in these two major T cell subsets (Fig. 4A).

Next, the degree of CD8^+^ or CD4^+^ T cell infiltration into the tumor was analysed by flow cytometry. Results suggest no significant differences in total numbers of infiltrated T cells in tumors from WT and Apelin KO mice (Fig. 4B). However, the distribution of CD8^+^ and CD4^+^ T cells detected by immunofluorescence staining in different regions of the tumor revealed that higher numbers of CD8^+^ T cells accumulated in the center of tumors from WT mice than Apelin-KO mice (Fig. 4C,D). Similarly, in the osmotic pump Apelin infusion models, both CD8^+^ and CD4^+^ T cell accumulation in the central areas were strongly enhanced by [Pyr^1^]Apelin-13 (Fig. 4E,F). This suggests that Apelin-induced mature vessel networks provide a better infiltrating route for T cells.

Immune cell infiltration into tumors is controlled by a coordinated arrangement of sequential steps, eg. for T cells, rolling, adhesion, and transmigration, extravasation and infiltration into target areas^20^. Crucial factors associated with these steps are adhesive interactions between T cells and endothelial cell surfaces, and integration of chemokine-mediated signalling. Based on these considerations, we evaluated the expression by ECs of adhesion molecules such as VE-Cad, ICAM-1, VCAM-1, and E-selectin, that are involved in T cell homing^11^. However, the expression of these molecules was found not to be different in the presence or absence of Apelin in the tumors (Fig. S3). This indicates that the enhanced accumulation of CD8^+^ and CD4^+^ T cells in central tumor areas might be regulated through a chemotactic interaction.

### Apelin induces production of CCL8 by vascular endothelial cells

**Figure 5:**
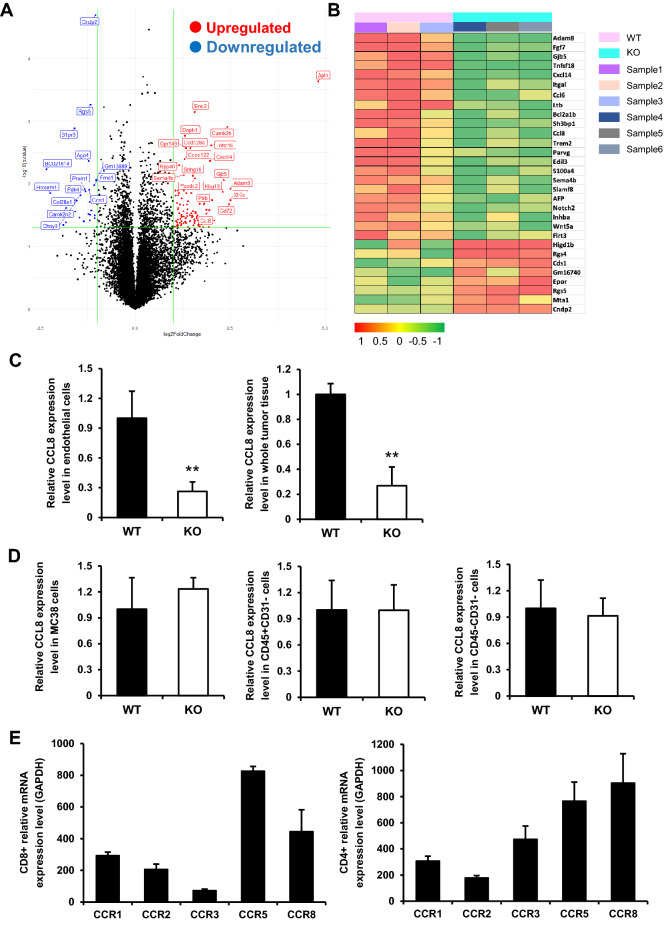
Global transcript expression levels of tumor endothelial cells analyzed by RNA sequencing. (**A**) Volcano plot of RNA-seq transcriptome data depicting gene expression patterns for ECs isolated from WT relative to Apelin-KO mice tumors at day 19 (n = 3 for each group). Volcano plot was illustrated by R 4.0.4 with packages ggplot2 and ggrepel. (**B**) Heatmap of 30 highly changed genes in ECs from WT or Apelin-KO mice bearing tumors (n = 3 for each group). Heatmap was drawn with log2 FPKM values. (**C**) qRT-PCR showing CCL8 expression levels in ECs and whole tumor tissue normalized to WT mouse tumor CCL8 expression. (**D**) qRT-PCR showing CCL8 expression levels in MC38 cancer cells, CD45^+^ CD31^−^ immune cells and CD45^−^ CD31^−^ stromal cells, respectively, normalized to WT mouse tumor CCL8 expression. (**E**) qRT-PCR of CCR expression levels in CD8^+^ and CD4^+^ T cells. The error bars indicate mean ± SD and all data were analyzed by two-sided Student's t-test. **p < 0.01.

To determine how Apelin recruits CD8^+^ and CD4^+^ T cells into central regions of the tumor, global transcriptomics for tumor ECs from WT or Apelin-KO mouse tumors was performed by RNA-sequencing analysis (Fig. 5A). It was found that 94 genes were expressed more than twofold higher in ECs from WT relative to Apelin KO-mouse tumors. The heat map shows 30 genes with different expression levels (Fig. 5B). Subsequent qRT-PCR analyses confirmed that among these, CCL8 was significantly more highly expressed in WT ECs than in Apelin-KO ECs (Fig. 5C). However, CCL8 expression was not different between WT and Apelin-KO mice in other tumor-associated cell types, including the MC38 cancer cells, CD45^+^ CD31^−^ immune cells and CD45^−^ CD31^−^ stromal cells (Fig. 5D). We performed immunohistochemistry to test the expression of CCL8 protein in WT ECs and KO ECs, but there was no obvious difference between them in this respect. The reason for this may be that CCL8 is a secreted protein and thus difficult to detect in the sparse cytoplasm of endothelial cells (Fig. S4). On the other hand, we also performed in vitro experiments using HUVEC by adding [Pry^1^]Apelin-13 and VEGF for 6 h. Thereafter, we analysed CCL8 mRNA expression and found that Apelin indeed induced CCL8 expression in vitro (Fig. S5). CCL8 is a CC chemokine that utilizes multiple cellular receptors, CCRs, to attract and activate circulating leukocytes^21^. Therefore, expression of CCRs was also assessed in intratumoral CD8^+^ and CD4^+^ T cells. Both T cell subsets expressed high levels of the CCR5 and CCR8 receptors for CCL8, higher than other CCL receptors (Fig. 5E). We also performed qRT-PCR for CCL5 expression in tumors and ECs (Fig. S6) and again found no differences between WT and Apelin-KO mice. On the basis of these results, we hypothesize that the Apelin-induced CCL8 secreted by ECs provides a chemotactic signal to CD8^+^ and CD4^+^ T cells and promotes T cell extravasation from vessels and infiltration into the tumor.

## Discussion

In our previous study, overexpression of Apelin significantly inhibited colon 26 tumor growth in mouse subcutaneous inoculation models^4^. However, the mechanism by which Apelin alone inhibits tumor growth had not been determined. In the present study, we demonstrate that similar inhibitory effects on tumor growth are also observed in MC38 and LLC s.c. tumor inoculation models (Fig. 3). On the other hand, several lines of evidence indicate that Apelin is often over-expressed in tumors, and it has been suggested that this factor may induce tumor growth because its expression correlates with tumor malignancy^3,22–25^. In some mouse tumor models, it has been reported that Apelin induces tumor growth; however, survival rate in those tumor models was not evaluated. On the other hand, Mastrella and colleagues recently reported that reduced apelin expression led to accelerated glioblastoma cell invasion^26^. In that paper, the authors showed that APLNR-positive tumor cells started to disseminate when Apelin levels were reduced both in in vitro and in vivo*,* and that data from patient samples were consistent with this in humans. In our MC38 and LLC tumor models, Apelin also inhibited tumor growth. Based on these results, it is possible that the real function of Apelin in tumor progression might be completely different to the current paradigm, namely that the up-regulation of Apelin in tumors may suggest a compensatory mechanism to inhibit tumor proliferation and migration. In any event, these differences in different tumor models indicate that Apelin has diverse functions in tumor formation depending on the intratumoral conditions. Vascular formation patterns and intra-tumoral immune cell types, and other factors, might vary under different intratumoral conditions.

Apelin deficiency led to formation of vascular tubes with narrower diameters and to a shortened vascular tree relative to tumors in WT mice, while [Pyr^1^]Apelin-13 peptide infusion resulted in blood vessels with larger diameters, although total vessel length was shorter (Fig. 3). The reason for this difference has not been determined in the present study. We hypothesize that this effect of exogenous Apelin causing enlarged blood vessel formation would be due to its inducing proliferation of ECs in WT mouse tumors. However, Apelin-stimulated EC proliferation is already saturated in the tumor microenvironment and excessive amounts of exogenous Apelin rather induce cell–cell aggregation of ECs but not their further proliferation, as reported by Kidoya et al.^17^. Therefore, enlargement of blood vessels is the end result of exogenously added Apelin in WT mice. In order to clarify the effect of exogenous Apelin, further analysis of the mechanism by which Apelin induces enlargement of vascular structures in the tumor microenvironment is required.

Although we found that CD8^+^ and CD4^+^ T cells accumulate more in the central areas of the tumor in the present study, whether Apelin-induced accumulation of immune cells is specific for these T cells or whether other cell types also accumulate is still not clear. However, we do know that the distribution of macrophages throughout the tumor was not different in the presence or absence of Apelin. Moreover, regulatory T cells were also not affected (data not shown). At present, the reason why Apelin affects CD4^+^ or CD8^+^ T cell distribution, but not other types of immune cells, has not been clarified. One possibility may be because MC38 colon cancer is intrinsically a type of tumor more sensitive to immune checkpoint blockade with a higher infiltration of T cells^27^. It is also possible that the combination of chemokines induced by Apelin, especially including CCL8, may strongly affect CD4^+^ and CD8^+^ T cell distribution. Further analysis is required to evaluate this possibility.

Although CCL8 is a stromal cell-induced chemokine, no significant difference in these cells between WT and Apelin-KO mice was seen in this study (data not shown). Compared to other cell types, EC are thought to contribute to the difference of CCL8 expression. However, how Apelin regulates the secretion of CCL8 in ECs is also still unknown. So far, there appear to be no studies showing that Apelin/APJ signalling correlates with CCL8 protein production. Therefore, further investigation is required to determine the relationships between CCL8 and CD8^+^ and CD4^+^ homing at the molecular level, although a CCL8 gradient might be expected to be a key factor^28^.

The outcome of cancer-immune interactions is based on a large number of parameters such as tumor “foreigness”, general immune status, absence of expression of the checkpoint molecule PD-L1, and immune cell infiltration^29–31^. In a previous publication, it has been reported that Apelin overexpression combined with αGalCer/DCs treatment improves iNKT cell infiltration, which leads to a tumor-inhibitory effect^4^. Therefore, in addition to CD8^+^ and CD4^+^ T cell infiltration, other factors also need to be taken into consideration in future work.

Combined with other anti-cancer drugs, normalization of the tumor vasculature using anti-angiogenic inhibitors can balance angiogenesis stimulators and inhibitors to block tumor growth^32–36^. However, the clinical benefits of these drugs have remained suboptimal, possibly because exploiting the concept of tumor vascular normalization has not been clearly recognized by clinicians, so anti-angiogenic drugs are not often employed for the specific purpose of such vascular normalization. Hence, tumor vessel normalization provides a new perspective on tumor inhibition, and increasing numbers of non-angiogenic inhibitors are considered for use in inducing vessel maturation. However, as a tumor vessel maturation inducer, exploiting only the Apelin/APJ axis alone may not be able to cure cancer completely. Combinations with other chemotherapeutics should be considered to achieve better results in clinic.

## Methods

### Mice and cell lines

Wild-type (WT) C57BL/6 mice were purchased from Japan SLC (Shizuoka, Japan). Apelin-knockout (KO) mice on the C57BL/6 background were generated as described previously^17^. APJ-knockout (KO) mice were gifts from Prof. Fukamizu^37^. All mice were used at the age of 8–10 weeks. Animals were housed in environmentally-controlled rooms in the animal experimentation facility at Osaka University. All experiments were performed in compliance with the laws and institutional guidelines of Osaka University Committee for Animal and Recombinant DNA Experiments. This study was approved by Osaka University Research Ethics Committee (Approval number 4062). This research was carried out in compliance with the Animal Research: Reporting of In Vivo Experiments (ARRIVE) guidelines.

MC38 (mouse colon carcinoma cell line) and LLC (mouse Lewis lung carcinoma cell line) purchased from the Riken cell bank (Tsukuba, Japan) were maintained in DMEM (Sigma-Aldrich, St. Louis, MO) supplemented with 10% fetal bovine serum (FBS, Sigma) and 1% penicillin/streptomycin (100 U/ml, PS; Life Technologies, Tokyo, Japan). HUVECs purchased from Kurabo (Osaka, Japan) were cultured in Humedia EG2 (Kurabo). They were starved and then stimulated with VEGF-A (20 ng/ml, PeproTech, Rocky Hill, NJ, USA) for 24 h after which [Pry^1^]Apelin-13 (1 µg/ml) was added for 6 h.

### qRT-PCR analysis

Briefly, total RNA was extracted from cells using RNeasy-plus mini kits (Qiagen, Hilden, Germany) and reverse-transcribed using the PrimeScript RT reagent kit (Takara) according to the manufacturer’s protocol. Real-time PCR analysis was performed using Platinum SYBR Green qPCR SuperMix-UDC (Invitrogen) and an Mx3000p QPCR System (Agilent, Santa Clara, CA, USA). Primers were as follows: mouse Apelin, sense 5′-GTG CCC TCC CGG TGC CGG TCT CT-3′, anti-sense 5′-GAG ACC ACG CCA TTA GAG GAA CT-3′; mouse APJ, sense 5′-CCA CTG TGG GCC ACT TAT ACC-3′, anti-sense 5′-CAG CCT TAG CCG AGC ATT G-3′; mouse CCL8, sense 5′-ACG CTA GCC TTC ACT CCA AAA-3′, anti-sense 5′-GTG ACT GGA GCC TTA TCT GG-3′; mouse CCR1, sense 5′-CTC ATG CAG CAT AGG AGG CTT-3′, anti-sense 5′-ACA TGG CAT CAC CAA AAA TCC A-3′; mouse CCR2, sense 5′-ATC CAC GGC ATA CTA TCA ACA TC-3′, anti-sense 5′-CAA GGC TCA CCA TCA TCG TAG-3′; mouse CCR3, sense 5′-TCG AGC CCG AAC TGT GAC T-3′, anti-sense 5′-CCT CTG GAT AGC GAG GAC TG-3′; mouse CCR5, sense 5′-TTT TCA AGG GTC AGT TCC GAC-3′, anti-sense 5′-GGA AGA CCA TCA TGT TAC CCA C-3′; mouse CCR8, sense 5′-ACG TCA CGA TGA CCG ACT ACT-3′, anti-sense 5′-CCC AGC ACA AAC AAG ACG C-3′; mouse CCL5, sense 5′-ATA TGG CTC GGA CAC CAC TC-3′, anti-sense 5′-GCA CTT GCT GCT GGT GTA GA-3′; mouse VE-cadherin, sense 5′-TGC TCA CGG ACA AGA TCA GC-3′, anti-sense 5′-CAT TCT GGC GGT TCA CGT TG-3′; mouse ICAM-1, sense 5′-GTG ATG CTC AGG TAT CCA TCC A-3′, anti-sense 5′-CAC AGT TCT CAA AGC ACA GC G-3′; mouse VCAM-1, sense 5′-GCC CAC TAA ACG CGA AGG T-3′, anti-sense 5′-ACT GGG TAA ATG TCT GGA GCC-3′; mouse E-selectin, sense 5′-ATG GAA GCC TGA ACT GCT CC-3′, anti-sense 5′-CAT TCA ACC ACA TGG CAG GC-3′; human CCL8, sense 5′-GAT GAA GGT TTC TGC AGC GC, anti-sense 5′-AAT CTG GCT GAG CAA GTC CC; mouse GAPDH, sense 5′-AGG TCG GTG TGA ACG GAT TTG-3′, anti-sense 5′-TGT AGA CCA TGT AGT TGA GGT CA-3′. Results were normalized to GAPDH using the comparative threshold cycle method.

### In vivo tumor subcutaneous inoculation model

MC38 or LLC cancer cells (1 × 10^6^ cells per mouse in 100 µl PBS) were inoculated subcutaneously into WT C57BL/6 or Apelin-KO mice (8 to 10 weeks of age). Tumors were dissected 18–20 days after implantation. In bone marrow (BM) transplantation models, BM cells were obtained from the tibias and femurs of WT or APJ-KO donor mice (C57BL/6 background, 8 weeks of age). WT C57BL/6 mice were transplanted intravenously with 1 × 10^6^ donor whole BM cells after lethal irradiation (10.0 Gy). Eight weeks after BM transplantation, MC38 cancer cells (1 × 10^6^ per mouse in 100 µL PBS) were inoculated subcutaneously, and tumor tissues were dissected 19 days after implantation. Tumor volumes were measured with callipers every 2–3 days and calculated as follows: width × width × length × 0.52.

### Osmotic pump transplantation model

1 × 10^6^ MC38 cancer cells per mouse in 100 µl PBS were subcutaneously inoculated into WT or Apelin-KO C57BL/6 mice as described above. ALZET osmotic pumps (model 1002, Cupertino, CA, USA) were loaded with either 100 µl [Pyr^1^]Apelin-13 (Becham, Bubendorf, Switzerland) dissolved in normal saline at a concentration of 8 mg/ml, or with 100 µl normal saline alone. Four days after cancer cell implantation, prepared pumps were implanted subcutaneously on the same side as the tumors, delivering continuous [Pyr^1^]Apelin-13 or saline infusions for 2 weeks. A daily [Pyr^1^]Apelin-13 dose of 2 mg/kg body weight per day was chosen based on previous in vivo work by Parry et al.

### Flow cytometry

Isolation of cells from mouse tumors, cell-surface antigen staining, and flow cytometry were performed as described previously^38^. Fluorescence-labelled anti-mouse CD45 (BioLegend), anti-mouse CD31 (BD Pharmingen), anti-mouse CD8 (eBioscience), and anti-mouse CD4 (eBioscience) were used. The stained cells were analysed and sorted by a FACSAria (BD Bioscience) and data were analysed using FlowJo Software (Treestar Software, San Carlos, California, USA). Dead cells were excluded by propidium iodide staining or by using the two-dimensional profile of the forward-versus-side scatter.

### Immunostaining analysis

Tissue fixation procedures were the same as those described previously^39^. Briefly, tumors were fixed with 4% paraformaldehyde (PFA) in PBS overnight, treated with 15%, 30% sucrose in PBS subsequently and embedded in OCT compound (Sakura Finetek, Tokyo, Japan). Frozen blocks were sectioned at 10 μm. The following primary antibodies were used: rat anti-mouse CD31 (BD Pharmingen), hamster anti-mouse CD31 (Merck Millipore, Darmstadt, Germany), rat anti-mouse CD8a (BioLegend), rat anti-mouse CD4-PE (eBiosciences), rat anti-mouse CCL8 (R&D Systems, Minneapolis, MN). Anti-rat Alexa Fluor 488 (Jackson ImmunoResearch Laboratories) and Cy3-conjugated anti-hamster IgG (Jackson ImmunoResearch) were used as the secondary antibody.

### Evaluation of hypoxia in tumors

To measure hypoxic status in tumor tissues, Hypoxyprobe-1 (60 mg/kg, i.p.; Hypoxyprobe, Burlington, MA, USA) was injected 2 h before tissues were harvested. Tumor sections were stained using the anti-Hypoxyprobe antibody, following the manufacturer’s instructions.

### RNA-seq analysis

Methods for RNA-seq were previously described^40^. Briefly, Total RNA of tumor endothelial cells was extracted from cells using RNeasy-plus mini kits. Library preparation was performed using a TruSeq stranded mRNA sample prep kit (Illumina, San Diego, CA) according to the manufacturer’s protocol. All Sequencing libraries were performed on an Illumina Hiseq 2500 platform in a 75-base single-end mode and Illumina Casava 1.8.2 software was used for base-calling. Sequenced reads were mapped to the mouse reference genome sequences (mm10) using TopHat v2.0.13 in combination with Bowtie2 ver. 2.2.3 and SAMtools ver. 0.1.19. The fragments per kilobase of transcript per million mapped reads (FPKMs) value was calculated using Cufflinks version 2.2.1. We uploaded to the GEO repository (https://www.ncbi.nlm.nih.gov/geo/, GEO accession number; GSE178681).

### Microscopy

Sections were examined by conventional microscopy (DM5500 B; Leica, Wetzlar, Germany) and images were acquired with a digital camera (DFC500; Leica). Images were processed using imageJ software (available at https://imagej.nih.gov/ij/). All images shown are representative of more than five independent experiments.

### Statistical analysis

All data are presented as the means ± s.d. and were analysed by two-sided Student's t-test. A P value of < 0.05 was considered statistically significant.

## Supplementary Information

Supplementary Information.
