# Supplementary material for: Endothelial cell-derived Apelin inhibits tumor growth by altering immune cell localization
Source: Sci Rep. 2021 Jul 7;11:14047. doi: 10.1038/s41598-021-93619-5 (PMC8263715; doi:10.1038/s41598-021-93619-5)
Supplement: Supplementary file 1 — Supplementary Information. [file 41598_2021_93619_MOESM1_ESM.pdf]

Supplemental Information

Endothelial cell-derived Apelin inhibits tumor growth by altering immune cell localization

Liuying Hu[1, #], Yumiko Hayashi[1, #], Hiroyasu Kidoya[1], Nobuyuki Takakura[1,2,3, *]

[1] Department of Signal Transduction, Research Institute for Microbial Diseases, Osaka University, Suita, Japan, [2]World Premier Institute Immunology Frontier Research Center, Osaka University, [3]Integrated Frontier Research for Medical Science Division, Institute for Open and Transdisciplinary Research Initiatives (OTRI), Osaka University,

[*]To whom correspondence should be addressed: ntakaku@biken.osaka-u.ac.jp

Figure S1

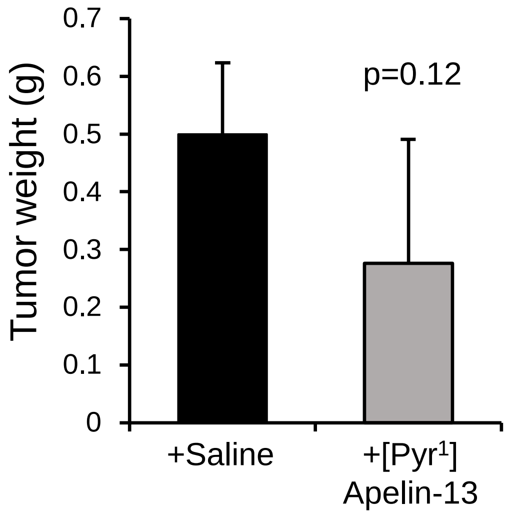

Figure S1. Tumor weight of MC38 cells subcutaneously inoculated into Apelin-KO mice receiving [Pyr^1]Apelin-13 or saline infusion using osmotic pumps (n=5 in each group). Data are mean ± SD and analyzed by two-sided Student's t-test.

Figure S2

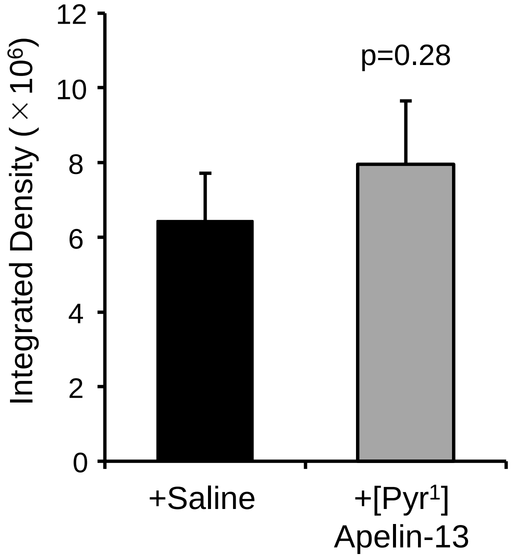

Figure S2. Total vessel density of tumors in WT + [Pry^{1}]Apelin-13 and WT + Saline (n=3 in each group). The error bars indicate mean ± SD and analysed by two-sided Student's t-test.

Figure S3

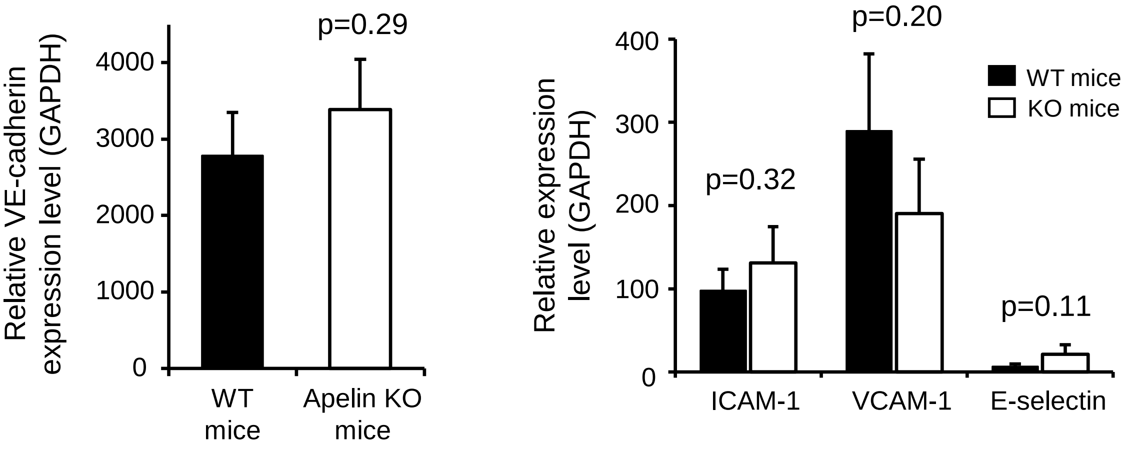

Figure S3. VE-cadherin, ICAM-1, VCAM-1, and E-selectin expression by qRT-PCR in tumor ECs in WT mice or Apelin-KO mice (n=3). Expression levels were normalized to GAPDH. The error bars indicate mean ± SD and all data were analysed by two-sided Student's t-test.

Figure S4

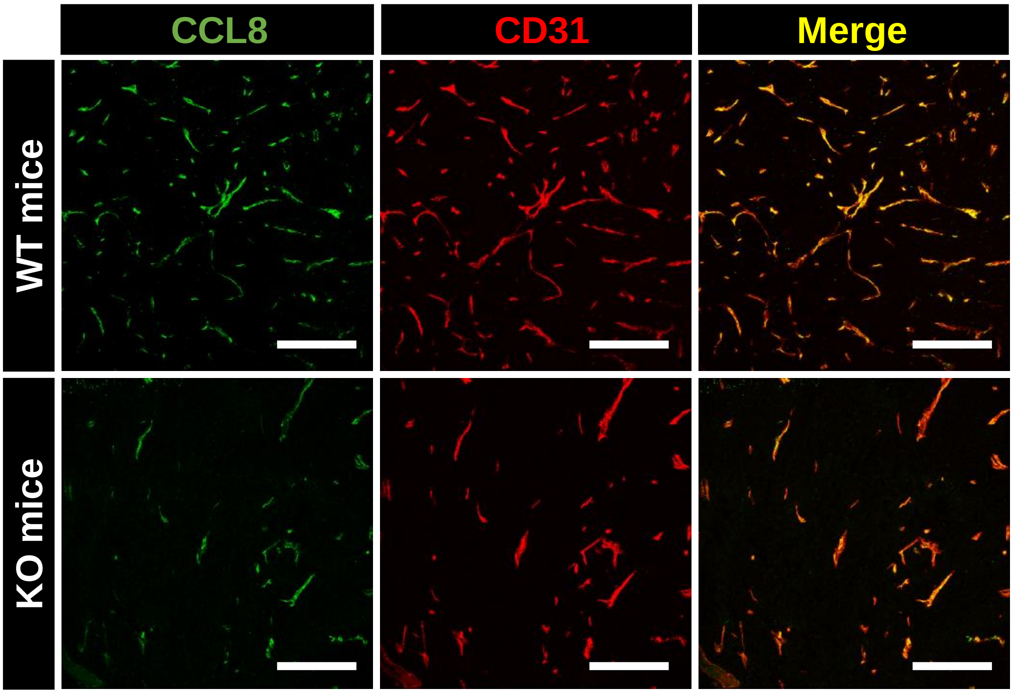

Figure S4. Representative images of MC38 tumors in WT mice or Apelin KO mice stained with anti-CCL8 (green) and CD31 (red) antibodies. Note that CCL8 protein expression was observed in endothelial cells; however, in this experiment, no obvious differences of CCL8 expression are detectable. Scale bars = 200 μm.

Figure S5

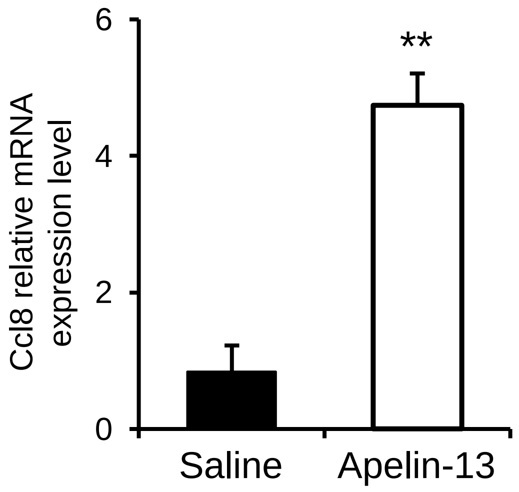

Figure S5. CCL8 expression level by qRT-PCR in HUVEC treated with Saline or [Pyr^1]Apelin-13 (1 µg/ml) for 6 hr after the addition of VEGF-A (20 ng/ml) (n=3). CCL8 expression levels were normalized to GAPDH. The error bars indicate mean ± SD and analyzed by two-sided Student's t-test. **p<0.01.

Figure S6

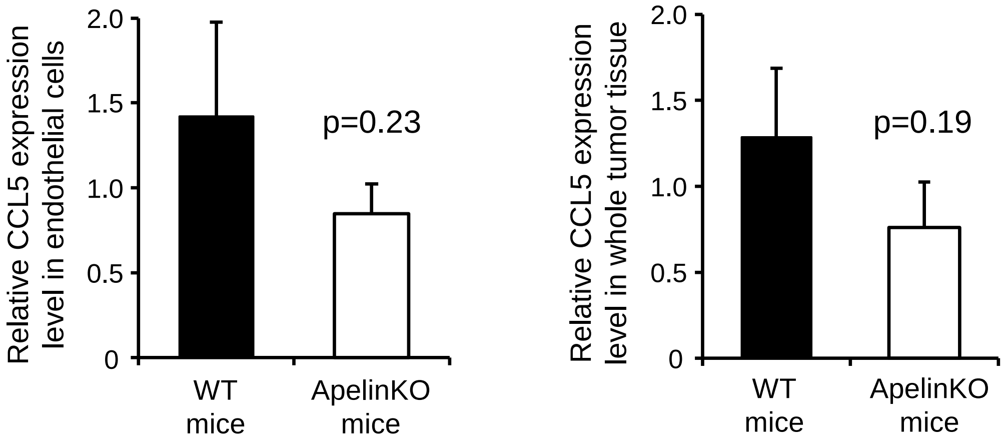

Figure S6. qRT-PCR of CCL5 expression levels in ECs and whole tumor tissue normalized to WT mouse tumor CCL5 expression (n=3). CCL5 expression levels were normalized to GAPDH. The error bars indicate mean ± SD and analyzed by two-sided Student's t-test.
